# Supplementary material for: Complexome profiling of the Chlamydomonas psb28 mutant reveals TEF5 as an early PSII assembly factor
Source: Plant Cell. 2025 Mar 17;37(6):koaf055. doi: 10.1093/plcell/koaf055 (PMC12164590; doi:10.1093/plcell/koaf055)
Supplement: koaf055_Supplementary_Data [file koaf055_supplementary_data.zip › psb28_interactive.zip!/plots/intensity/qualityPlots/peptides/_Cre17.g707900.t1.2.html]

### Cre17.g707900.t1.2

Trivial name:   
SeqCoverage [median]: 15.80  
Mass: 37.03 kDa  
  
 

| Sequence | MissedCleavage | Mass [Da] | wtIntensity | muIntensity | Score |
| --- | --- | --- | --- | --- | --- |
| AEAAVVER | 0 | 843.45 | 409.98 | 874.82 | 124.98 |
| ATAAAGAADATAK | 0 | 1088.55 | 358.07 | 1088.86 | 152.70 |
| ITEVHAQVSAQLPK | 0 | 1519.84 | 0.00 | 536.30 | 68.03 |
| LAEQAAK | 0 | 729.40 | 226.26 | 0.00 | 91.91 |
| VAELTQQLTAATTR | 0 | 1501.81 | 344.06 | 3411.10 | 143.42 |
|  |  |  | 1338.4 | 5911.1 |  |
